# How COVID-19 Pandemic Restrictions Affected Kuwaiti College Students’ Anthropometry, Lifestyle Behaviors, and Dietary Habits

**DOI:** 10.3390/nu15224773

**Published:** 2023-11-13

**Authors:** Ahmad R. Al-Haifi, Nayef Y. Bumaryoum, Balqees A. Al-Awadhi, Fahad A. Alammar, Bader N. Alkhalaf, Hazzaa M. Al-Hazzaa

**Affiliations:** 1 Department of Food and Nutrition Science, College of Health Sciences, The Public Authority for Applied Education & Training, Shuwaikh 70654, Kuwaitny.bumaryoum@paaet.edu.kw (N.Y.B.); b.alawadhi@paaet.edu.kw (B.A.A.-A.); 2 Department of Environmental Health, College of Health Sciences, The Public Authority for Applied Education & Training, Shuwaikh 70654, Kuwait; bn.alkhalaf@paaet.edu.kw; 3 Lifestyle and Health Research Center, Health Sciences Research Center, Princess Nourah Bint Abdulrahman University, Riyadh 11546, Saudi Arabia; halhazzaa@hotmail.com

**Keywords:** COVID-19 pandemic, college students, Kuwait, lifestyle changes, eating habits, physical activity, body weight, sleep patterns, academic achievement, gender differences

## Abstract

The COVID-19 pandemic and the measures implemented to control it have led to widespread lifestyle changes globally. While previous studies have explored these effects across different age groups, this research focuses uniquely on college students in Kuwait. A cross-sectional study (N = 1259) was conducted among college students using a validated online questionnaire covering various aspects, including demographics, academic performance, self-reported body weight and height, sleep duration, dietary habits, and physical activity. There were equal proportions of individuals reporting decreased, increased, or no change in body weight, BMI, and dietary habits due to COVID-19, with no significant gender differences observed. A higher decrease (44.3%) than increase (29.2%) occurred due to COVID-19 in the proportion of college students engaging in physical activity. Significant differences were found in the students’ responses to most of the eating behavior questions, concerning the changes attributable to the COVID-19 pandemic restrictions. Multivariable analysis showed significant interaction effects for gender by losing above 9 kg during the last 6 months in age (*p* = 0.037), total physical activity in METs-min/week (*p* = 0.048), and cake/donuts intake (*p* = 0.006). Logistic regression analysis, adjusted for age, revealed that a decrease in BMI was associated with an increase in daily vegetable intake (aOR = 1.531, *p* = 0.031), whereas increases in BMI were associated with an increased intake of sugar-sweetened drinks equal to or above 4 days/week (aOR = 1.551, *p* = 0.032) and increased chocolates/candy intake equal to or above 4 days/week (aOR = 1.792, *p* = 0.037). It was concluded that, in response to a major epidemic, such as COVID-19, college students, as a population, are susceptible to significant changes in lifestyle and eating behaviors that can impact their health and well-being. Future risks for college students’ health can be reduced through appropriate lifestyle interventions.

## 1. Introduction

The coronavirus disease 2019 (COVID-19) was declared a pandemic by the World Health Organization in March 2020 [1]. As of 26 August 2023, it had resulted in a staggering global toll of 6.9 million deaths and 770 million reported cases [1]. To mitigate the rapid spread of the virus and its associated morbidity and mortality rates, governments worldwide swiftly implemented a range of measures, including total or partial lockdowns, quarantines, social distancing mandates, and the closure of schools and workplaces [2].

While the impact of such measures was felt across all age groups, children and adolescents emerged as particularly vulnerable to the mental health challenges brought about by the pandemic [3,4,5]. The disruptions to their usual routines, physical activity, and social interactions, coupled with the stress and uncertainty of the situation, placed students at an increased risk for various psychological issues. It is reasonable to hypothesize that these psychological challenges may have contributed to the changes observed in lifestyle, eating behaviors, and BMI, which are the focus of this research [6]. Numerous studies have reported a surge in negative emotional states among young adults, including heightened levels of anxiety, loneliness, stress, anger, irritability, psychological distress, and depressive symptoms [7,8]. Furthermore, there has been a notable increase in body mass index (BMI) and higher rates of overweight among this demographic [9]. Individuals with pre-existing eating disorders have been especially impacted, with a concerning 41% experiencing a reactivation of their symptoms during lockdowns [8].

Across the globe, the COVID-19 pandemic has influenced many aspects of people’s lives such as their health, body weight, education, social life, and lifestyles behaviors [10,11,12]. Studies conducted in neighboring countries, such as Jordan [9] and Saudi Arabia [13,14], observed similar trends, further emphasizing the regional relevance and importance of this issue. As a consequence of COVID-19, physical activity was impacted with a drastic reduction in exercising, walking, and bicycling for transportation and leisure [15,16]. In addition, as a result of at-home confinement, sedentary behavior frequently replaced time spent in physical activity [16].

College students are generally sociable human beings, and, therefore, the period of social isolation during the COVID-19 lockdowns is considered a major factor that can impact their lifestyle and eating behaviors [12,15,17]. In addition, the closure of universities and the introduction of online teaching definitely disrupted their usual daily routines, added more anxiety and stress to their lives, and may have negatively influenced their mental health and well-being [18]. Hence, it was particularly important to maintain a healthy lifestyle and well-adjusted eating habits during this critical period of the pandemic.

Furthermore, the impacts of COVID-19 on lifestyle behaviors may vary from country to country due to differences in the cultural, social, economic, and types of healthcare systems [11,12,19]. In addition, some recent studies observed gender-related health risk differences as a result of COVID-19 pandemic restrictions [20,21].

In the State of Kuwait, there is limited research examining the impacts of the COVID-19 pandemic on body weight and lifestyle behaviors, including physical activity levels, screen time, sleep, and dietary habits. Therefore, the aim of this study was to evaluate the impact of the COVID-19 pandemic restrictions on self-reported body weight, sleep duration, lifestyle behaviors, eating habits, and academic achievement among college students in Kuwait. Moreover, our study aimed to identify and compare gender differences between male and female students in the measured variables, providing insights into how the pandemic affected each group. Previous research indicated that the pandemic’s effects may vary based on gender [20,21].

## 2. Materials and Methods

### 2.1. Sample and Design

This cross-sectional study was conducted in Kuwait over a 4-month period from November 2020 to February 2021. Ethical approval was secured from the Institutional Review Board (IRB) at the College of Health Sciences in Kuwait. The sample size was calculated with the assumption that the population proportion would yield the maximum possible sample size required (proportion = 0.50), with a confidence level of 95% and a margin of error of 4%. An additional 20% of participants were added to account for non-responders or missing data. The total sample size for each gender was calculated to be 480, with 960 students in total.

An online questionnaire was answered by college students attending various colleges across the university (under the Public Authority of Applied Education and Training (PAAET) in Kuwait). The questionnaire was distributed to students electronically, given the restrictions imposed by the pandemic. Participants were provided with clear instructions on how to complete the questionnaire online. The recruitment process involved contacting the colleges’ administrations to post the recruitment letter along with the online questionnaire on the colleges’ internet sites; teachers who were teaching online were asked by the colleges to advise interested students to participate in the survey. Before completing the questionnaire, students were informed of the study objectives and procedures and consented to participate in the study. The inclusion criteria were college students (males and females), above 17 years old, without having any disease that prevents him/her from freely moving, and that the participant was free of any nutritional disorders.

### 2.2. Questionnaire and Data Collection

The questionnaire was composed of demographic variables (gender and age,) self-reported body weight (kg) and height (cm), marital status, governorate name, the student’s college, the college grade point average (GPA), the high school grade, as well as the lifestyle behaviors items. The lifestyle behaviors questionnaire included questions on physical activity, screen time, sleep duration, and dietary habits. The lifestyle questionnaire was previously validated for adolescents and young people and was previously published [22,23,24,25]. The physical activity questionnaire collected extensive data about the time and frequency of engaging in a variety of activities related to light-, moderate-, and vigorous-intensity physical activities in different domains (leisure-type, sports, transportation like walking, and household activities). Then, the activity was assigned a metabolic equivalent of task (MET) based on the Compendium of Physical Activities. Physical activity then was reported either in minutes per week or as activity energy expenditure in METs-min/week.

Body mass index (BMI) was calculated from weight and height data. Students were also asked about their weight and height both before and during the COVID-19 pandemic. This enabled us to assess changes in BMI and body weight during the pandemic period. The current data used in the present study were part of a prior research project [26].

### 2.3. Statistical Analysis

Data were entered into an SPSS data file, checked, cleaned, and analyzed using the SPSS program, version 22 (IBM, Chicago, IL, USA). Descriptive statistics were calculated and presented as means and standard deviations (SD) or proportions. Cross tabulation with chi-square tests was used to calculate the proportion (%) of changes due to COVID-19 (before versus during the pandemic) in anthropometric measurements, lifestyle factors, and eating behaviors among the college students relative to gender. We also used multivariable analysis to test the differences in the characteristics of those college students who lost 9 kg or more during the last 6 months of COVID-19 relative to gender (the eating-related items contained a question asking about this issue). Bonferroni tests were used for testing between-subjects’ effects. In addition, logistic regression analysis was used to test the associations of selected demographic and lifestyle behaviors relative to changes in BMI status due to COVID-19 among the college students, while adjusted for age. An alpha level of ≤0.05 was used as the level of significance.

## 3. Results

The anthropometric characteristics of the participating college students, categorized by gender, are summarized in Table 1. The study included a total of 1259 participants, comprising 54.1% females, with a mean age (SD) of 21.5 (3.1) years. No significant age differences relative to gender were observed (*p* = 0.133). However, significant gender differences were noted in body weight, height, and body mass index (BMI), with males exhibiting higher values (*p* < 0.001). Additionally, more than 10% of the participants were married. The prevalence of overweight or obesity stood at 42%, with males having a significantly higher rate than females (*p* < 0.001).

Table 2 presents the proportions (%) of changes in anthropometric and lifestyle factors among Kuwaiti college students due to COVID-19, categorized by gender. Notably, there were relatively equal proportions of individuals reporting decreased, increased, or no change in body weight, BMI, and dietary habits due to COVID-19, with no significant gender-based differences observed in the proportions of changes (decrease, increase, or no change) in body weight (*p* = 0.376), BMI (*p* = 0.417), or dietary habits (*p* = 0.133). However, more decrease (44.3%) than increase (29.2%) occurred due to COVID-19 in the proportion of Kuwaiti college students engaging in physical activity, with no gender difference (*p* = 0.767). Table 2 provides a detailed breakdown of these changes.

The proportional changes (%) in eating behaviors among college students resulting from the COVID-19 pandemic and associated restrictions are outlined in Appendix A. As indicated in the table, significant differences were found in the students’ responses to most of the eating behavior questions (26 items) concerning the changes attributable to the COVID-19 pandemic restrictions. However, there were no significant differences in the following questions relative to students’ responses (not included in Table 3): I feel that those around me would like me to eat more (*p* = 0.224), I throw up after eating foods (*p* = 0.574), I take more time than others when eating my meals (*p* = 0.478), I feel that those around me push me to eat (*p* = 0.582), Foods occupy my time and thinking (*p* = 0.358), I like to have empty stomach (*p* = 0.060), and I feel the urge to throw up after eating meals (*p* = 0.962).

The results of the multivariable analysis, displayed in Table 3, focus on the characteristics of college students who reported losing 9 kg or more during the last 6 months of the COVID-19 pandemic. Significant interaction effects were observed for gender by losing above 9 kg during the last 6 months in age (*p* = 0.037), total physical activity in METs-min/week (*p* = 0.048), and cake/donuts intake (*p* = 0.006). Additionally, the significant, main effects of losing above 9 kg during the last 6 months were noted in several variables including body weight (*p* < 0.001), BMI (*p* < 0.001), total physical activity (*p* < 0.001), moderate-intensity physical activity (*p* < 0.001), vigorous-intensity physical activity (*p* < 0.001), as well as intakes of vegetables (*p* = 0.004), fruits (*p* < 0.001), sugar-sweetened drinks (*p* = 0.023), fast foods (*p* = 0.001), French fries/potato chips (*p* = 0.006), chocolates/candy (*p* = 0.003), and energy drinks (*p* = 0.012). Furthermore, college GPA was also significantly associated with this category (*p* = 0.010).

Table 4 shows the results of logistic regression analysis, adjusted for age, which explores the relationship between selected demographic and lifestyle behaviors and changes in BMI status due to the COVID-19 pandemic among college students. A decrease in BMI of 53% was associated with an increase in daily vegetable intake (aOR = 1.531, *p* = 0.031), whereas increases in BMI of 55% and 79% were associated, respectively, with an increased intake of sugar-sweetened drinks equal to or above 4 days/week (aOR = 1.551, *p* = 0.032) and increased chocolates/candy intake of equal to or above 4 days/week (aOR = 1.792, *p* = 0.037).

## 4. Discussion

The COVID-19 pandemic and the subsequent measures, including lockdowns and social distancing, had profound effects on individuals’ lifestyles and behaviors across the globe. While previous research predominantly focused on specific age groups such as children, adolescents, and adults, our study uniquely examined the impact of the COVID-19 pandemic on college students’ body weight, lifestyle behaviors, and eating habits.

### 4.1. Impact on Body Weight and BMI

Although nearly one-third of the students in our study experienced an increase in body weight and BMI due to the lockdowns and quarantine measures, the overall findings did not show significant differences among those who increased, decreased, or maintained their body weight. However, the COVID-19 lockdown resulted in weight gain among Jordanian children and adolescents [9,27]. Additionally, among Malaysian university students, there was a weight gain of about 3.4 kg after the first month of the COVID-19 lockdown [17]. Similarly, two systematic reviews and meta-analyses examining nearly 100 studies indicated that during the COVID-19 pandemic lockdowns there were increases amounting to 11.1–72.4% in body weight among young adults in these studies [28,29]. It was believed that the main cause of increased body weight among individuals during COVID-19 lockdowns was the move to online education, which involved higher screen time and long sitting hours along with increased food consumption and snacking [17,28,29,30]. Also, with lower physical activity levels, lower diet quality, and more frequent overeating, there was a higher chance of increased BMI [31].

### 4.2. Changes in Lifestyle Behaviors and Eating Habits

The present findings regarding changes in lifestyle behaviors and eating habits align with those observed in various studies conducted during the pandemic [27,32,33,34]. Furthermore, our research highlights significant changes in eating habits. Increased consumption of unhealthy foods and snacks was prevalent among our participants, which is in line with findings from various international and regional studies [32,33,34]. Moreover, the findings of the current study appeared consistent with similar findings worldwide indicating that the COVID-19 pandemic had profound effects on lifestyle and eating habits. A study carried out on Portuguese university students showed that healthier eating habits were adopted by the students during the lockdown period compared to a typical semester period. Such healthier behaviors included reduced meal delivery, a lower intake of sugar, salt, and fast food, as well as higher engagement in physical activities and more consumption of fruits, vegetables, and legumes [35]. The decreased physical activity levels observed in our study, especially among females, are consistent with global trends during the pandemic [27,32,33,34]. Nevertheless, contrary to many other studies’ findings, studies from Italy [36] and Portugal [10] found a greater adherence to engaging in physical activity during the lockdown period.

The above changes, due to the COVID-19 lockdown, can be attributed to various factors such as increased stress, boredom, and greater access to unhealthy foods at home [37]. The absence of social interaction, especially among young college students, could lead to increased stress, anxiety, and even depression [18]; such feelings of isolation might elicit emotional eating in order to relieve adverse feelings [38].

Notably, our study indicates that most participants reported changes in eating habits, underscoring the widespread impact of the pandemic on dietary behaviors. Lockdown can bring about some healthy eating behaviors. Eating habits of college students from Tabriz (Iran) were characterized by an increase in a regular meal pattern and balanced diet, milk or its products, and egg or meat in the diet, as well as by a reduction in fast food intake, fried food, potato chips, and sugar-sweetened drinks. However, the students reported increases in weight gain, screen and sitting time, and sleep duration, as well as declined physical activity and higher stress or anxiety levels during the COVID-19 pandemic [37]. Similar studies carried out in Italy, Spain, Romania, and Latin America showed some improvement in dietary habits including an increased intake of fruits, vegetables, yogurt, legumes, and eggs during the lockdown period [36,39,40,41].

The findings of the present study do not agree with results found in a study conducted on adults from the UK, which showed a higher number of participants reporting more negative changes in their eating habits, including more frequent snacking, during the COVID-19 confinement compared to the pre-lockdown period [30]. In addition, a Portuguese study, conducted on adults, observed that the majority of the participants consumed more foods rich in fat and salt and sugar-sweetened beverages during the restriction period compared to pre-pandemic [10].

### 4.3. Sleep Patterns and Academic Achievement

Sleep duration, an essential component of overall health, also experienced alterations during the pandemic. We observed that a substantial number of students reported shifts in their sleep patterns, with many experiencing later bedtimes and wake times. This pattern mirrors the changes in daily routines brought about by remote learning and reduced social commitments, as seen in other studies [32,33]. Despite that, our findings did not show any significant changes in sleep duration relative to a decrease or increase in weight or BMI. However, the interaction of sleep duration, BMI, and lifestyle factors is quite complex, as exhibited in our current study.

Our findings on academic achievement reveal a mixed picture. While some students reported improvements in academic performance due to the flexibility of online learning, others experienced challenges related to motivation and concentration. These variations may be attributed to individual differences in adapting to remote learning environments as well as the overall impact of the pandemic on mental health and stress levels [7,8,34].

### 4.4. Gender Differences

Aside from the pandemic, it is well recognized that women on average report more physical and mental unhealthy days per year than men [42]. However, reports indicate that women are at a reduced risk of severe disease and death with SARS-CoV-2 infection than men [43,44]. Gender differences in our study show that there were greater changes among females in their body weight, physical activity, and sleep patterns. These gender differences align with the observations made by Al Hourani et al., who noted significant increases in body weight among females [27]. In addition, recent studies observed some differences in gender-related health risks resulting from COVID-19 pandemic restrictions [20,21]. Further, the psychological abuse of women as a type of violence reportedly increased significantly during the lockdown [45].

### 4.5. Strengths, Limitations, and Future Research

The current research strengths included a large sample of male and female college students from a variety of colleges and the use of a validated lifestyle questionnaire. However, our study is not without limitations. One notable limitation is the use of self-reported data for variables such as body weight and height, which may introduce reporting bias due to a social desirability effect. Additionally, there is a small chance that the sample may not be fully representative of all Kuwaiti college students, although we targeted all college students when recruiting participants.

Future research could involve more objective measures, such as clinical assessments of body composition and the use of accelerometers for the assessment of physical activity, sedentary behaviors, and sleep, to mitigate the limitations associated with self-reported data. Longitudinal studies are also warranted to assess the long-term consequences of the observed lifestyle changes and their impact on the health and well-being of college students.

## 5. Conclusions

As the result of a major epidemic, such as COVID-19 restrictions, college students appear to be susceptible to significant changes in lifestyle and eating behaviors that can impact their health and well-being. To the best of our knowledge, the current research is the first study that reported about the effects of the COVID-19 pandemic on body weight, lifestyle behaviors, and eating habits among college students in the State of Kuwait using a fairly large and representative sample. The COVID-19 pandemic has profoundly impacted the lives of college students in Kuwait, manifesting in significant changes in anthropometry, lifestyle behaviors, and dietary habits. This study, despite its local focus, contributes to the growing body of literature on the pandemic’s effects, emphasizing the need for tailored interventions and ongoing research. The pandemic’s influence on body weight and BMI is evident, with nearly 30% of college students showing an increase in body weight and BMI. Reduced physical activity levels, dietary changes, disrupted sleep patterns, and variations in academic performance further underscore the comprehensive impact.

Future risks for college students’ health can be reduced through appropriate lifestyle interventions, hence, promoting healthier lifestyle behaviors and dietary habits among college students during crises. These interventions should be multidimensional, addressing not only the physical health but also the psychosocial well-being of students. While our research provides valuable insights into the specific context of Kuwaiti college students, more extensive investigations are warranted to explore the underlying mechanisms and long-term implications of these changes. Collaboration among policymakers, educators, healthcare professionals, and parents is essential to foster a healthier environment for college students amid and beyond the pandemic.

## Figures and Tables

**Table 1:** Descriptive characteristics of the participating college students.

Variable	All N = 1259	Male N = 577	Female N = 682	*p*-Value *
Age (year)	21.5 ± 3.1	21.7 ± 3.1	21.4 ± 3.1	0.133
Body weight (kg)	69.5 ± 19.7	78.7 ± 21.2	61.6 ± 14.3	<0.001
Body height (cm)	165.9 ± 9.8	174.1 ± 6.7	158.9 ± 6.0	<0.001
Body mass index (kg/m^2^)	25.1 ± 6.3	25.9 ± 7.0	24.3 ± 5.5	<0.001

Data are means ± standard deviations; * *t*-test for independent samples for the differences between males and females.

**Table 2:** Changes (%) in anthropometric and lifestyle factors due to COVID-19 among Kuwaiti college students.

Variable	Change (%) *	All N = 1259	Male N = 577	Female N = 682	*p*-Value **
Body weight	Decrease	34.3	34.7	34.0	0.376
	Increase	27.3	26.2	28.3	
	No change	38.4	39.1	37.7	
BMI	Decrease	34.3	34.7	34.1	0.417
	Increase	27.2	26.1	28.2	
	No change	38.5	39.2	37.7	
Physical activity levels	Decrease	44.3	43.2	45.2	0.767
	Increase	29.2	29.6	28.8	
	No change	26.5	27.2	26.0	
Dietary habits	Deteriorated	27.9	25.8	29.6	0.133
	Improved	38.9	41.8	36.5	
	No change	33.2	32.4	33.9	

* The changes indicate from before to during COVID-19. ** Chi-square tests for the differences in proportions between males and females. Explanation: “Deteriorated” indicates that students reported worsening of dietary habits. “Improved” means students reported better dietary habits. “No Change” implies no significant change in dietary habits.

**Table 3:** Multivariable analysis of the characteristics of those Kuwaiti college students who lost 9 kg or more during the last 6 months of COVID-19 relative to gender (n = 1188).

Variable	Gender	Losing ≥ 9 kg during the Last 6 Months		*p*-Value *
		Never	Yes	
Age (years)	Male	21.4 ± 2.9	22.1 ± 3.3	Losing 9 kg: 0.086 Gender: 0.051 Losing 9 kg by gender interaction: 0.037
	Female	21.4 ± 3.2	21.4 ± 3.0	
	All	21.4 ± 3.1	21.7 ± 3.1	
Body weight (kg)	Male	75.5 ± 20.9	84.7 ± 20.5	Losing 9 kg: <0.001 Gender: <0.001 Losing 9 kg by gender interaction: 0.052
	Female	59.9 ± 13.8	64.5 ± 14.5	
	All	66.7 ± 18.9	74.2 ± 20.3	
BMI (kg/m^2^)	Male	25.1 ± 6.5	27.6 ± 6.4	Losing 9 kg: <0.001 Gender: <0.001 Losing 9 kg by gender interaction: 0.643
	Female	23.6 ± 5.2	25.7 ± 5.5	
	All	24.3 ± 5.8	26.6 ± 6.0	
Screen time (hours/day)	Male	4.6 ± 3.1	4.9 ± 3.7	Losing 9 kg: 0.988 Gender: <0.001 Losing 9 kg by gender interaction: 0.152
	Female	5.8 ± 3.8	5.5 ± 4.2	
	All	5.3 ± 3.6	5.2 ± 4.0	
Sleep duration (hours/night)	Male	7.4 ± 1.6	7.2 ± 1.6	Losing 9 kg: 0.841 Gender: 0.051 Losing 9 kg by gender interaction: 0.228
	Female	7.4 ± 1.6	7.5 ± 1.7	
	All	7.4 ± 1.6	7.4 ± 1.6	
Total physical activity (METs-min/week)	Male	2376.7 ± 2935.7	2700.9 ± 2832.1	Losing 9 kg: <0.001 Gender: <0.001 Losing 9 kg by gender interaction: 0.048
	Female	1424.7 ± 1876.2	2350.6 ± 2416.4	
	All	1840.6 ± 2441.8	2519.2 ± 2627.4	
Moderate-intensity physical activity (METs-min/week)	Male	579.4 ± 892.5	720.5 ± 1014.6	Losing 9 kg: <0.001 Gender: <0.001 Losing 9 kg by gender interaction: 0.116
	Female	782.6 ± 1001.4	1118.8 ± 1442.3	
	All	693.8 ± 960.1	927.1 ± 1154.1	
Vigorous-intensity physical activity (METs-min/week)	Male	1797.4 ± 2405.8	1980.5 ± 2195.4	Losing 9 kg: <0.001 Gender: <0.001 Losing 9 kg by gender interaction: 0.078
	Female	642.1 ± 1233.9	1231.7 ± 1611.9	
	All	1146.8 ± 1926.0	1592.1 ± 1949.1	
Breakfast intake (day/week)	Male	4.0 ± 2.7	3.9 ± 2.7	Losing 9 kg: 0.671 Gender: 0.799 Losing 9 kg by gender interaction: 0.090
	Female	3.8 ± 2.8	4.2 ± 2.9	
	All	3.9 ± 2.7	4.0 ± 2.8	
Vegetables intake (day/week)	Male	4.0 ± 2.5	4.5 ± 2.4	Losing 9 kg: 0.004 Gender: 0.992 Losing 9 kg by gender interaction: 0.578
	Female	4.0 ± 2.5	4.4 ± 2.6	
	All	4.0 ± 2.5	4.4 ± 2.5	
Fruit intake (day/week)	Male	3.0 ± 2.3	3.5 ± 2.5	Losing 9 kg: <0.001 Gender: 0.913 Losing 9 kg by gender interaction: 0.927
	Female	3.0 ± 2.4	3.5 ± 2.4	
	All	3.0 ± 2.4	3.5 ± 2.4	
Milk/dairy products intake (day/week)	Male	4.3 ± 2.5	4.5 ± 2.5	Losing 9 kg: 0.514 Gender: 0.225 Losing 9 kg by gender interaction: 0.833
	Female	4.2 ± 2.6	4.3 ± 2.5	
	All	4.3 ± 2.5	4.4 ± 2.5	
Sugar-sweetened drink intake (day/week)	Male	4.0 ± 2.6	3.7 ± 2.7	Losing 9 kg: 0.023 Gender: <0.001 Losing 9 kg by gender interaction: 0.847
	Female	3.5 ± 2.5	3.1 ± 26	
	All	3.7 ± 2.6	3.4 ± 2.7	
Fast food intake (day/week)	Male	3.4 ± 2.1	3.1 ± 2.1	Losing 9 kg: 0.001 Gender: <0.001 Losing 9 kg by gender interaction: 0.381
	Female	3.1 ± 2.1	2.6 ± 1.9	
	All	3.2 ± 2.1	2.8 ± 2.0	
French fries/potato chips intake (day/week)	Male	2.9 ± 2.1	2.8 ± 2.1	Losing 9 kg: 0.006 Gender: 0.434 Losing 9 kg by gender interaction: 0.071
	Female	3.1 ± 2.2	2.5 ± 2.0	
	All	3.0 ± 2.1	2.6 ± 2.1	
Cake/donuts intake (day/week)	Male	2.3 ± 2.1	2.4 ± 2.1	Losing 9 kg: 0.101 Gender: 0.001 Losing 9 kg by gender interaction: 0.006
	Female	3.1 ± 2.4	2.5 ± 2.0	
	All	2.7 ± 2.3	2.5 ± 2.1	
Chocolates/candy intake (day/week)	Male	3.2 ± 2.4	3.0 ± 2.4	Losing 9 kg: 0.003 Gender: 0.002 Losing 9 kg by gender interaction: 0.074
	Female	3.9 ± 2.4	3.2 ± 2.3	
	All	3.6 ± 2.4	3.1 ± 2.3	
Energy drinks intake (day/week)	Male	1.5 ± 2.2	2.0 ± 2.4	Losing 9 kg: 0.012 Gender: 0.002 Losing 9 kg by gender interaction: 0.364
	Female	1.2 ± 2.1	1.5 ± 2.3	
	All	1.4 ± 2.1	1.7 ± 2.3	
High school grade (%)	Male	75.1 ± 8.4	75.4 ± 9.2	Losing 9 kg: 0.369 Gender: 0.171 Losing 9 kg by gender interaction: 0.146
	Female	75.2 ± 8.9	73.9 ± 9.1	
	All	75.1 ± 8.7	74.6 ± 9.2	
College GPA	Male	3.00 ± 0.56	2.95 ± 0.60	Losing 9 kg: 0.010 Gender: 0.054 Losing 9 kg by gender interaction: 0.279
	Female	3.12 ± 0.61	2.98 ± 0.61	
	All	3.07 ± 0.60	2.97 ± 0.61	

Data are means and standard deviations. * Wilks’ lambda *p* values for multivariable tests: age < 0.001; gender < 0.001; losing 9 kg < 0.001; and gender by losing 9 kg interactions <0.001.

**Table 4:** Logistic regression analysis of lifestyle behaviors relative to changes in BMI status due to COVID-19.

Variable	Changes in BMI Status *			
	aOR	(95% CI)	SEE	*p*-Value
Decrease in BMI				
Age (younger age = ref)	1.024	0.967–1.084	0.029	0.424
Gender (female = ref)	0.891	0.633–1.253	0.1174	0.506
Marital status (married = ref)	1.257	0.704–2.244	0.296	0.439
Screen time (high screen time = ref)	1.00			
Low screen time	0.990	0.696–1.410	0.180	0.957
Sleep duration (sufficient sleep = ref)	1.00			
Insufficient sleep	0.995	0.702–1.412	0.178	0.979
Physical activity (low active = ref)	1.00			
High active	1.008	0.721–1.409	0.171	0.962
Breakfast intake (non-daily intake = ref)	1.00			
Daily intake	1.038	0.723–1.491	0.185	0.838
Vegetables intake (non-daily intake = ref)	1.00			
Daily intake	1.531	1.039–2.258	0.198	0.031
Fruit intake (non-daily intake = ref)	1.00			
Daily intake	1.332	0.832–2.135	0.240	0.233
Milk/dairy products intake (non-daily intake = ref)	1.00			
Daily intake	1.009	0.693–1.467	0.191	0.965
Sugar-sweetened drink intake (<4 days/week = ref)	1.00			
≤ 4 days/week	0.798	0.553–1.150	0.187	0.226
Fast food intake (<4 days/week = ref)	1.00			
≥4 days/week	1.092	0.706–1.689	0.223	0.693
French fries/potato chips intake (<4 days/week = ref)	1.00			
≥4 days/week	0.802	0.513–1.252	0.227	0.331
Cake/donuts intake (<4 days/week = ref)	1.00			
≥4 days/week	0.874	0.515–1.484	0.270	0.618
Chocolates/candy intake (<4 days/week = ref)	1.00			
≥4 days/week	1.247	0.777–2.001	0.241	0.360
Energy drinks intake (<4 days/week = ref)	1.00			
≥4 days/week	0.738	0.453–1.202	0.249	0.222
Increase in BMI				
Age (younger age = ref)	1.011	0.952–1.074	0.031	0.717
Gender (female = ref)	0.855	0.600–1.218	0.181	0.385
Marital status (married = ref)	1.639	0.867–3.097	0.325	0.128
Screen time (high screen time = ref)	1.00			
Low screen time	0.855	0.593–1.233	0.187	0.401
Sleep duration (sufficient sleep = ref)	1.00			
Insufficient sleep	1.184	0.826–1.597	0.184	0.357
Physical activity (low active = ref)	1.00			
High active	0.745	0.524–1.058	0.179	0.100
Breakfast intake (non-daily intake = ref)	1.00			
Daily intake	0.827	0.572–1.196	0.188	0.314
Vegetables intake (non-daily intake = ref)	1.00			
Daily intake	1.124	0.757–1.669	0.202	0.561
Fruit intake (non-daily intake = ref)	1.00			
Daily intake	0.851	0.541–1.339	0.231	0.486
Milk/dairy products intake (non-daily intake = ref)	1.00			
Daily intake	1.414	0.958–2.088	0.199	0.081
Sugar-sweetened drink intake (<4 days/week = ref)	1.00			
≥4 days/week	1.551	1.039–2.314	0.204	0.032
Fast food intake (<4 days/week = ref)	1.00			
≥4 days/week	1.368	0.845–2.216	0.246	0.203
French fries/potato chips intake (<4 days/week = ref)	1.00			
≥4 days/week	0.736	0.455–1.191	0.246	0.212
Cake/donuts intake (<4 days/week = ref)	1.00			
≥4 days/week	0.896	0.501–1.599	0.295	0.710
Chocolates/candy intake (<4 days/week = ref)	1.00			
≥4 days/week	1.792	1.036–3.103	0.280	0.037
Energy drinks intake (<4 days/week = ref)	1.00			
≥4 days/week	0.604	0.365–1.002	0.258	0.051

* No change was used as a reference category; aOR = adjusted odds ratio; CI = confidence interval; ref = reference category; SEE = standard error.

## Data Availability

Data are contained within the article.

## Appendix A

**Table A1:** Responses to questions related to eating behaviors as a result of changes in body weight due to COVID-19 among Kuwaiti college students.

Variable	Response Choice	Change in Weight Due to COVID-19 (%)			*p*-Value
		Decreased N = 432	Increased N = 344	No Change N = 483	
I am scared of being overweight	Never	12.5	16.3	29.4	<0.001
	Rarely	6.5	8.4	14.7	
	Occasionally	19.2	16.0	14.3	
	Mostly	22.0	24.7	17.6	
	Always	39.8	34.6	24.0	
I avoid eating if I am hungry	Never	27.8	18.6	31.1	0.001
	Rarely	20.8	17.2	20.1	
	Occasionally	26.9	34.6	22.8	
	Mostly	19.7	23.3	21.7	
	Always	4.9	6.4	4.3	
I have difficulty stopping eating	Never	26.6	35.8	49.1	<0.001
	Rarely	17.8	25.9	17.4	
	Occasionally	23.8	17.7	15.1	
	Mostly	15.8	13.1	13.0	
	Always	13.2	7.6	5.4	
I eat my meals as several small snacks	Never	39.8	28.5	31.7	0.005
	Rarely	18.3	18.9	17.8	
	Occasionally	21.1	20.1	23.4	
	Mostly	12.3	19.5	18.4	
	Always	8.6	13.1	8.7	
I know the calories of my eaten foods	Never	51.6	31.1	49.3	<0.001
	Rarely	14.4	14.0	12.6	
	Occasionally	13.9	18.9	12.8	
	Mostly	13.0	19.2	15.9	
	Always	7.2	16.9	9.3	
I avoid carbohydrate-rich foods (like bread, potato, and rice)	Never	42.4	33.4	45.3	0.003
	Rarely	16.9	16.0	14.7	
	Occasionally	20.6	20.3	15.9	
	Mostly	14.6	19.8	18.4	
	Always	5.6	10.5	5.6	
I feel guilty after eating	Never	32.2	32.6	55.1	<0.001
	Rarely	13.4	13.4	12.0	
	Occasionally	18.8	22.7	11.0	
	Mostly	16.0	17.2	12.2	
	Always	19.7	14.2	9.7	
I am obsessed with the desire to be thin	Never	16.4	20.3	44.3	<0.010
	Rarely	6.5	7.6	8.3	
	Occasionally	13.7	13.7	11.6	
	Mostly	15.0	17.7	13.9	
	Always	48.4	40.7	21.9	
I think about burning calories when doing exercise	Never	14.6	16.9	32.5	<0.001
	Rarely	7.9	4.4	6.6	
	Occasionally	12.0	11.0	13.5	
	Mostly	24.5	21.2	20.3	
	Always	41.0	46.5	27.1	
People think I am very skinny	Never	35.9	31.1	26.3	0.035
	Rarely	10.6	12.8	9.9	
	Occasionally	16.9	16.0	15.9	
	Mostly	19.0	19.8	22.6	
	Always	17.6	20.3	25.3	
I am obsessed with the idea that I have fats in my body	Never	14.1	15.7	30.6	<0.001
	Rarely	9.0	6.1	10.8	
	Occasionally	15.5	18.3	16.6	
	Mostly	22.2	23.3	18.2	
	Always	39.1	36.6	23.8	
I avoid foods with sugar	Never	29.2	18.0	33.5	<0.001
	Rarely	18.8	13.1	17.2	
	Occasionally	27.8	27.6	23.0	
	Mostly	17.6	25.6	19.5	
	Always	6.7	15.7	6.8	
I eat diet foods	Never	39.1	28.2	41.4	<0.001
	Rarely	16.7	14.5	16.8	
	Occasionally	24.1	21.2	19.0	
	Mostly	16.2	23.3	16.8	
	Always	3.9	12.8	6.0	
I feel that diet is controlling my life	Never	26.4	26.5	37.2	0.001
	Rarely	10.9	11.6	12.2	
	Occasionally	17.4	20.1	16.8	
	Mostly	25.2	19.5	19.9	
	Always	20.1	22.4	14.1	
I can control myself in front of foods	Never	20.8	11.9	24.2	0.001
	Rarely	11.1	11.9	10.8	
	Occasionally	25.0	21.5	19.7	
	Mostly	26.9	31.7	24.6	
	Always	16.2	23.0	20.7	
I do not feel comfortable after eating sweets	Never	30.3	27.0	34.2	0.007
	Rarely	14.6	13.4	15.3	
	Occasionally	22.0	20.3	18.2	
	Mostly	13.7	20.6	20.1	
	Always	19.4	18.6	12.2	
I practice certain dietary behaviors	Never	43.1	30.8	51.1	<0.001
	Rarely	14.6	13.1	12.2	
	Occasionally	19.2	15.7	14.1	
	Mostly	16.0	24.1	14.1	
	Always	7.2	16.3	8.5	
I enjoy eating new fatty foods	Never	5.2	5.6	6.8	0.020
	Rarely	16.0	21.8	14.7	
	Occasionally	21.5	21.8	25.5	
	Mostly	25.7	21.2	22.2	
	Always	21.8	14.8	20.5	
